# Two endoplasmic reticulum proteins (calnexin and calreticulin) are involved in innate immunity in Chinese mitten crab (*Eriocheir sinensis*)

**DOI:** 10.1038/srep27578

**Published:** 2016-06-09

**Authors:** Ying Huang, Kaimin Hui, Min Jin, Shaowu Yin, Wen Wang, Qian Ren

**Affiliations:** 1 Jiangsu Key Laboratory for Biodiversity & Biotechnology and Jiangsu Key Laboratory for Aquatic Crustacean Diseases, College of Life Sciences, Nanjing Normal University, Nanjing 210046, China; 2 State Key Laboratory Breeding Base of Marine Genetic Resource, Third Institute of Oceanography, SOA, Xiamen 361005, China; 3 Co-Innovation Center for Marine Bio-Industry Technology of Jiangsu Province, Lianyungang, Jiangsu 222005, PR China

## Abstract

Calnexin (Cnx) and calreticulin (Crt), which are important chaperones in the endoplasmic reticulum (ER), participate in the folding and quality control of client proteins. *Cnx* and *Crt* identified from Chinese mitten crab (*Eriocheir sinensis*) are designated as *EsCnx* and *EsCrt*, respectively. *EsCnx* and *EsCrt* are expressed in the hemocyte, hepatopancrea, gill, and intestine at the mRNA and protein level. Immunofluorescence analysis indicated that EsCnx and EsCRT are located in the ER. Moreover, the mRNA and protein expression levels of *EsCnx* and *EsCrt* were altered by challenge with lipopolysaccharides (LPS), peptidoglycans (PGN), *Staphyloccocus aureus*, and *Vibrio parahaemolyticus*. Recombinant EsCnx and EsCrt (rEsCnx and rEsCrt, respectively) proteins can bind to various Gram-positive and Gram-negative bacteria, as well as to different polysaccharides (LPS and PGN). rEsCnx and rEsCrt assisted in the clearance of *V. parahaemolyticus in vivo*, and the clearance efficiency was impaired after silencing of *EsCnx* and *EsCrt*. Our results suggest that the two ER proteins are involved in anti-bacterial immunity in *E. sinensis*.

Chaperones perform critical roles in various physiological processes, such as in promotion of cell survival, irreversible aggregation of newly synthesized proteins, prevention of misfolding, maintenance of the unfolded conformation of newly synthesized polypeptides, binding to non-native proteins during cellular stress responses, assembly of large proteins and multiprotein complexes, intracellular signaling, transcription, and differentiation^1^,^2^,^3^. Calnexin (Cnx) and calreticulin (Crt) are related molecular chaperone proteins localized in the lumen of the endoplasmic reticulum (ER); these proteins are the most extensively studied glycoprotein-specific chaperones^4^. The ER is a dynamic organelle in cells and plays a wide array of functions in the synthesis of membrane and secretory proteins, as well in regulating protein folding, post-translational modification of membrane-associated proteins, secretion, and degradation^5^,^6^. This organelle provides an oxidizing environment that, along with chaperone and co-chaperone proteins, induces the formation of disulfide bonds, appropriate folding of nascent glycoproteins, and prevention of improper folding of proteins^7^. As marker proteins, Cnx and Crt preferentially interact with non-native proteins through N-linked oligosaccharide moieties to form relatively stable complexes in the ER^8^,^9^; this interaction persists until the proteins are properly folded or until misfolded proteins are degraded^10^,^11^.

Cnx, a lectin chaperone that was first isolated from mouse, is transiently associated with partly folded MHC class I molecules^12^; Cnx was subsequently cloned from the liver cDNA library of humans in 1991^13^. Cnx is also associated with incomplete forms of MHC class I molecules, T-cell receptors, and membrane immunoglobulins^14^,^15^. In addition, Cnx, with a molecular mass of approximately 90 kDa, is a non-glycosylated type I membrane protein present in many species^16^. The protein structure of Cnx contains three functionally distinct domains: a large ER luminal domain, a transmembrane domain, and an ER-retention motif in the cytoplasmic tail^13^. The amino acid sequences of Cnx in plants and animals contain conserved regions in the middle parts and highly diverse regions in the N- and C-termini^17^. Cnx and its soluble homolog Crt constitute a dedicated maturation system, known as the Cnx/Crt cycle, which mainly promotes correct folding and oligomerization of newly synthesized glycoproteins before their export from the ER^18^,^19^. Furthermore, Cnx plays a role in Ca^2+^ regulation^20^, phagocytosis (a phylogenetically conserved process)^21^, and cell sensitivity to apoptosis in the ER^22^.

Crt, a highly conserved ER luminal resident protein, plays important roles in quality control, Ca^2+^ homeostasis, lectin-like chaperoning, gene regulation, cell adhesion, wound healing, cancer, and autoimmunity^23^,^24^, as well as in various oxidative stress responses to hydrogen peroxide, hypoxic injury, and iron overload^25^,^26^,^27^. Crt not only exists inside and outside of the ER lumen but also on the cell surface or extracellular matrix^28^,^29^. Crt was first isolated from the sarcoplasmic reticulum of rabbit muscle cells and identified as a high affinity Ca^2+^-binding protein^30^; subsequent isolation of this protein in mouse indicated that Crt contains the ER retention/retrieval signal KDEL (Lys-Asp-Glu-Leu) sequence at the C-terminus and is primary located in the lumen of the ER^31^. Numerous Crt genes have been isolated from diverse organisms^32^. In nearly all eukaryotes, Crt contains several structural and functional domains, including an N-domain, which is a protein-interacting domain, as well as proline-rich (P) and C domains, which interact with Ca^2+^ (refs 33,34). Crt regulates Ca^2+^ levels by controlling the signaling and homeostasis of this ion and acts as a protein-folding chaperone by forming a complex with the ER protein 57 (ERp57)^32^,^35^. In addition, Crt affects biological processes, including growth, reproduction, molting, the immune system, apoptosis, and stress responses^23^,^36^.

The roles of molecular chaperones in modulating immune functions have received increased research attention. In this study, *EsCnx* and *EsCrt* were identified from crabs, and their potential functions in innate immunity were investigated. Data obtained in this study will elucidate the roles of Cnx and Crt in crab innate immunity.

## Results

### cDNA cloning and sequence analysis

**Figure 1:**
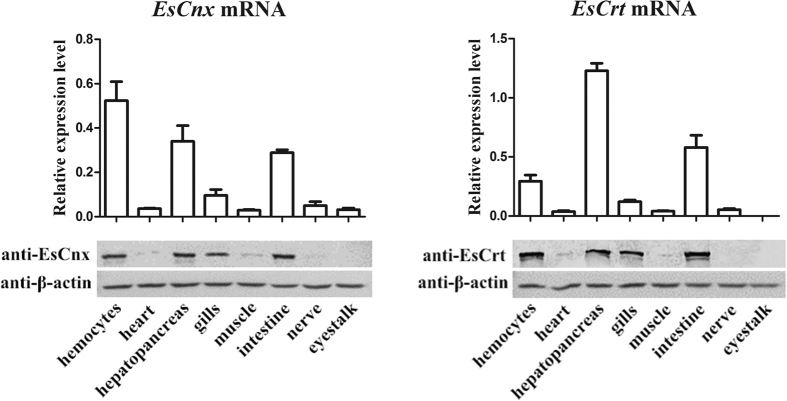
Tissue distributions of *EsCnx* and *EsCrt* at the mRNA level (above) revealed by SYBR Green qRT-PCR and protein expression level (below) revealed by western blot. Vertical bars represent mean ± S.E. (N = 5) for each tissue. Each bar represents the mean value from five determinations with standard error.

The 3175-bp full-length cDNA of *EsCnx* encodes a polypeptide of 598 amino acids; this peptide possesses a predicted molecular weight of 68 kDa and a theoretical isoelectric point of 4.50 (Fig. 1SA). *EsCrt* is 1741-bp long and encodes a protein of 406 amino acids; the calculated molecular weight and theoretical isoelectric point of EsCrt are 46.8 kDa and 4.29, respectively.

**Figure 2:**
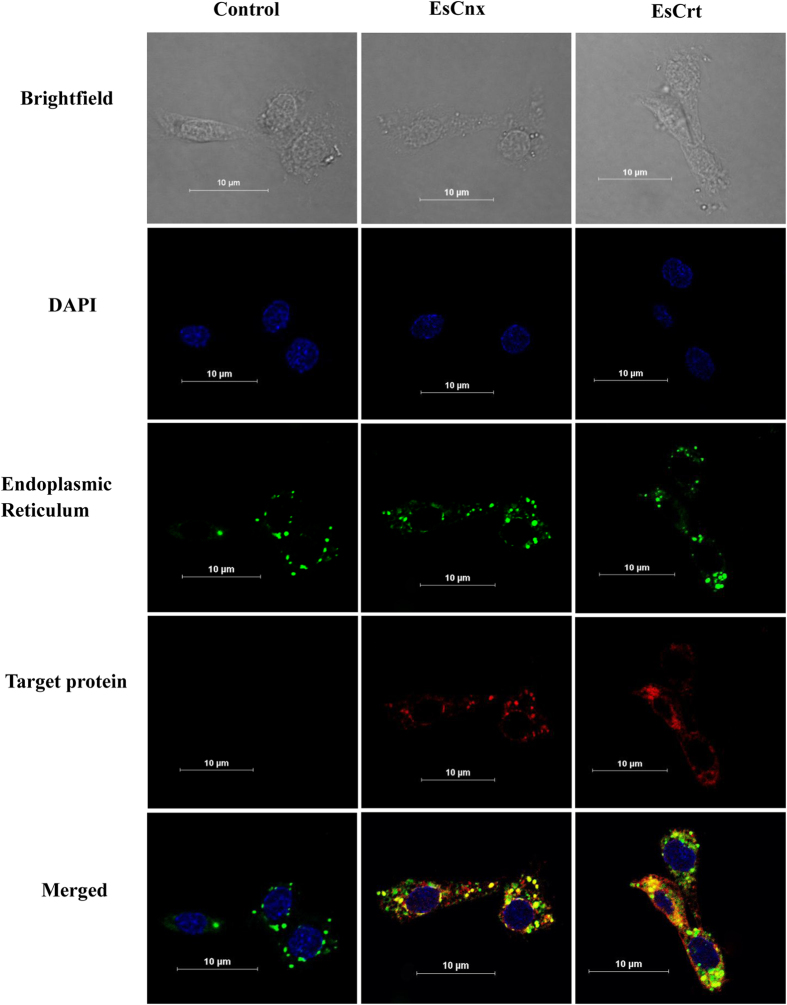
Intracellular localization of EsCrt and EsCnx in crab cells. Hemocytes were stained for EsCrt or EsCnx using rabbit anti-EsCrt antibody (1:1000) or anti-EsCnx (1:1000). DAPI was used to stain the nucleus. DiOC6(3) 3,3′-dihexyloxacarbocyanine iodide is a green fluorescent membrane dye used for staining the endoplasmic reticulum.

**Figure 3:**
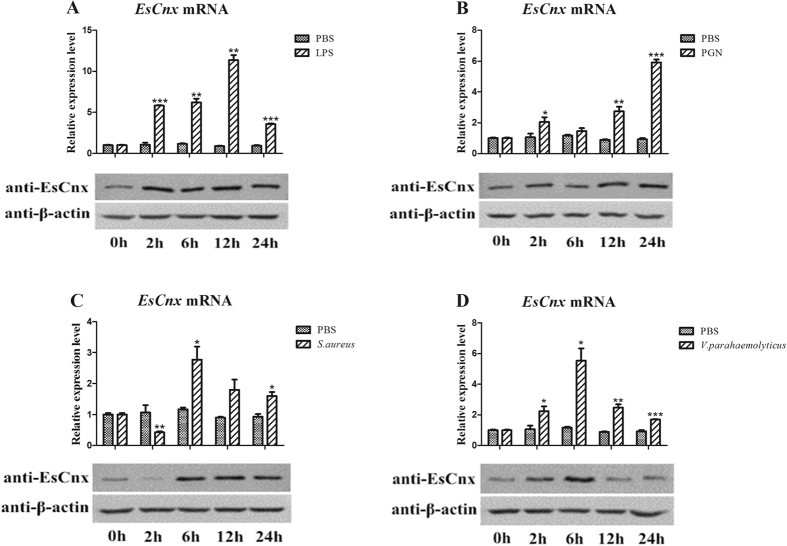
*EsCnx* mRNA and protein expression profile in hepatopancreas after LPS (**A**), PGN (**B**), *S. aureus* (**C**), and *V. parahaemolyticus* (**D**) challenge as measured by qRT-PCR and western blot. The GAPDH gene was used as internal control to calibrate the cDNA template of all samples. Vertical bars represent mean ± S.E. (N = 5). Each bar represents the mean value of five determinations with standard deviation. Asterisks indicate significant differences (*P < 0.05, **P < 0.01, ***P < 0.001) compared with the control. The β-actin was used as internal control to calibrate the protein template of all samples.

**Figure 4:**
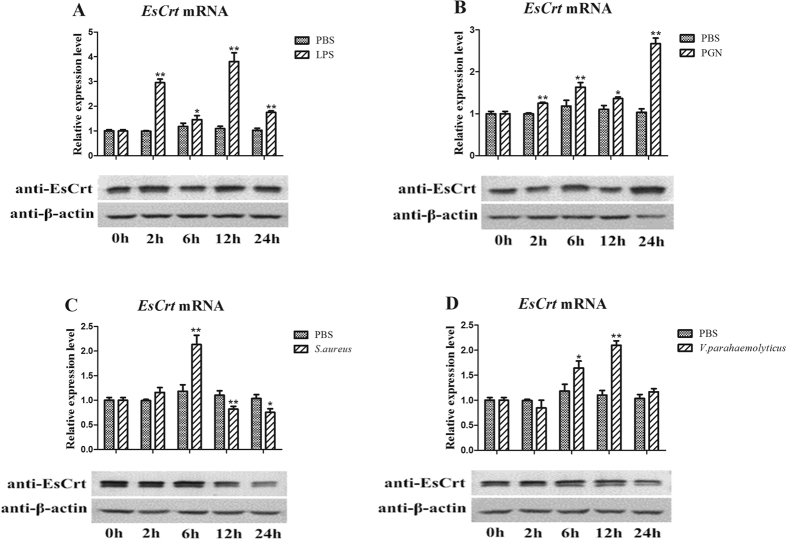
*EsCrt* mRNA and protein expression profile in the hepatopancreas after LPS (**A**), PGN (**B**), *S. aureus* (**C**), and *V. parahaemolyticus* (**D**) challenge as measured by qRT-PCR and western blot. GAPDH gene was used as an internal control to calibrate the cDNA template for all of the samples. Vertical bars represent mean ± S.E. (N = 5). Each bar represents the mean value of five determinations with standard deviation. Asterisks indicate significant differences (*P < 0.05, **P < 0.01, ***P < 0.001) compared with the values of control. The β-actin was used as internal control to calibrate the protein template of all samples.

BLASTP search and multiple sequence alignment showed that EsCnx exhibits sequence similarity to other reported crustacean Cnxs, such as 76% identity to that of *Marsupenaeus japonicas* (AIF71174.1) (Fig. 2SA). The deduced amino acid sequence of EsCrt shares significant homology with other known Crts, such as 92% similarity to that of *Scylla paramamosain* (AEN94572.1) (Fig. 2SB). The constructed phylogenetic tree showed that EsCnx, *M. japonicas* MjCnx, and *Penaeus monodon* PmCnx are clustered into one subgroup (Fig. 3S). Meanwhile, EsCrt and nine Crts from other crustaceans and *Culex quinquefasciatus* are clustered into one group (Fig. 4S).

### Tissue distribution of *EsCnx* and *EsCrt* and immunofluorescence assay

*EsCnx* and *EsCrt* mRNAs were highly expressed in the hemocyte, hepatopancrea, gill, and intestine (Fig. 1). The highest expression level of *EsCnx* was detected in hemocytes, followed by hepatopancreas and intestines. *EsCrt* was mainly expressed in the hepatopancreas, intestine, and hemocytes of healthy crabs. Western blot analysis showed the presence of EsCnx and EsCrt in hemocytes, hepatopancreas, gills, and intestine (Fig. 1). Furthermore, immunofluorescence assay combined with confocal microscopy analysis confirmed that EsCrt and EsCnx were located in the ER (Fig. 2).

### Analysis of protein and mRNA expression patterns of *EsCnx* and *EsCrt* after challenge with polysaccharides and microorganisms

When crabs were injected with lipopolysaccharides (LPS), the mRNA expression levels of *EsCnx* from 2 h to 12 h were significantly higher than those in the untreated control and then decreased at 24 h (Fig. 3A). After 2 h of peptidoglycan (PGN) challenge, *EsCnx* was initially upregulated, returned to its original level at 6 h, and then increased at 12 and 24 h (Fig. 3B). The transcript expression of *EsCnx* was initially downregulated 2 h after challenge with *Staphylococcus aureus*, reached the highest level at 6 h, and gradually decreased from 12 h to 24 h (Fig. 3C). After challenge with *Vibrio parahaemolyticus*, the *EsCnx* mRNA expression level gradually increased within 2 h, peaked at 6 h, and then decreased at 12 and 24 h (Fig. 3D). Moreover, the protein expression pattern of EsCnx was similar to that of mRNA expression upon challenge with LPS (Fig. 3A), PGN (Fig. 3B), *S. aureus* (Fig. 3C), and *V. parahaemolyticus* (Fig. 3D).

*EsCrt* was rapidly upregulated 2 h after LPS challenge, decreased at 6 h, increased at 12 h, and was finally downregulated again at 24 h (Fig. 4A). The EsCrt protein level did not change within 2–12 h after the LPS challenge (Fig. 4A). After 2–6 h of the PGN challenge, *EsCrt* mRNA and EsCrt protein expression levels were gradually upregulated, decreased at 12 h, and finally reached the highest levels (Fig. 4B). After 6 h of *S. aureus* challenge, *EsCrt* transcription peaked and then decreased from 12 h to 24 h (Fig. 4C). Upon *S. aureus* challenge, the protein expression pattern of EsCrt was similar to that of mRNA expression (Fig. 4C). After *V. parahaemolyticus* challenge, *EsCrt* expression increased from 6 h to 12 h and then recovered to the normal level (Fig. 4D). Furthermore, the protein expression level of EsCrt did not evidently change upon *V. parahaemolyticus* challenge (Fig. 4D).

### Expression and purification of recombinant proteins

**Figure 5:**
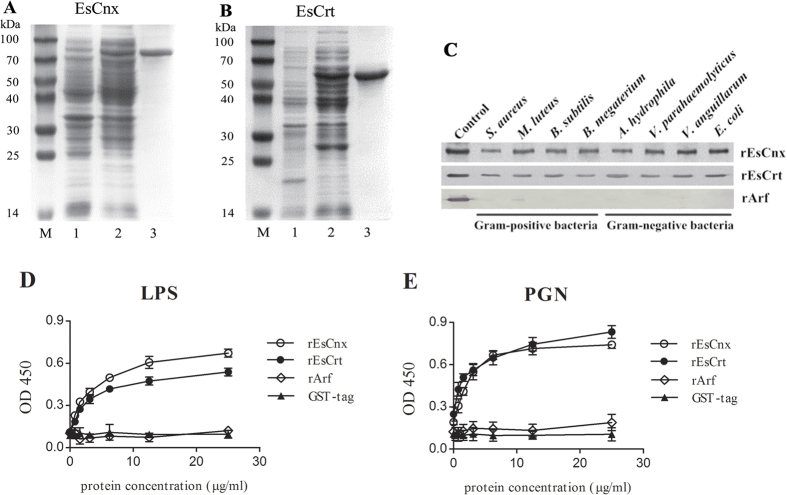
SDS-PAGE analysis of rEsCnx (**A**) and rEsCrt (**B**). Lane M: protein molecular weight standards (kDa); lane A1: negative control for rEsCnx (without induction); lane A2: IPTG-induced rEsCnx; lane A3: purified rEsCnx; lane B1: negative control for rEsCrt (without induction); lane B2: IPTG-induced rEsCrt; and lane B3: purified rEsCrt. (**C**) Direct binding of rEsCnx and rEsCrt proteins to microorganisms (*S. aureus*, *M. luteus*, *B. subtilis*, *B. megaterium*, *A. hydrophila*, *V. parahaemolyticus*, *V. anguillarum*, and *E. coli*). The microorganisms were incubated with rEsCnx or rEsCrt and then washed four times with TBS. ELISA assay was employed to detect the binding of rEsCnx (**D**) and rEsCrt (**E**) to LPS and PGN using antiserum against rEsCnx and rEsCrt. All assays were repeated three times.

Figure 5A shows a detected band with a molecular weight of approximately 70 kDa, which is consistent with the predicted molecular weight of the Crt region of EsCnx (MW: 43.1 kDa) with a 26-kDa GST tag. EsCrt bearing the GST tag was approximately 62 kDa (Fig. 5B).

### Microbial and carbohydrate binding of rEsCnx and rEsCrt

The bacterial binding assay showed that rEsCrt can bind to all tested microorganisms, including four kinds of Gram-positive bacteria (*S. aureus*, *M. luteus*, *B. subtilis*, and *Bacillus megaterium*) and four kinds of Gram-negative bacteria (*Aeromonas hydrophila*, *V. parahaemolyticus*, *Vibrio anguillarum*, and *Escherichia coli*). rEsCnx weakly bound to *S. aureus* and *A. hydrophila* but strongly bound to the six other microorganisms (Fig. 5C). No visible binding was detected in the negative control.

rEsCnx and rEsCrt demonstrated binding activity to PGN or LPS. The binding activity of rEsCnx to LPS was higher than that of rEsCrt; whereas the binding activity of rEsCrt to PGN was higher than that of rEsCnx. The binding ability of the proteins was found to be dose dependent (Fig. 5D,E). For the control, rArf and GST proteins did not bind to LPS or PGN.

### Effect of rEsCnx and rEsCrt on the clearance of *V. parahaemolyticus*

**Figure 6 f6:**
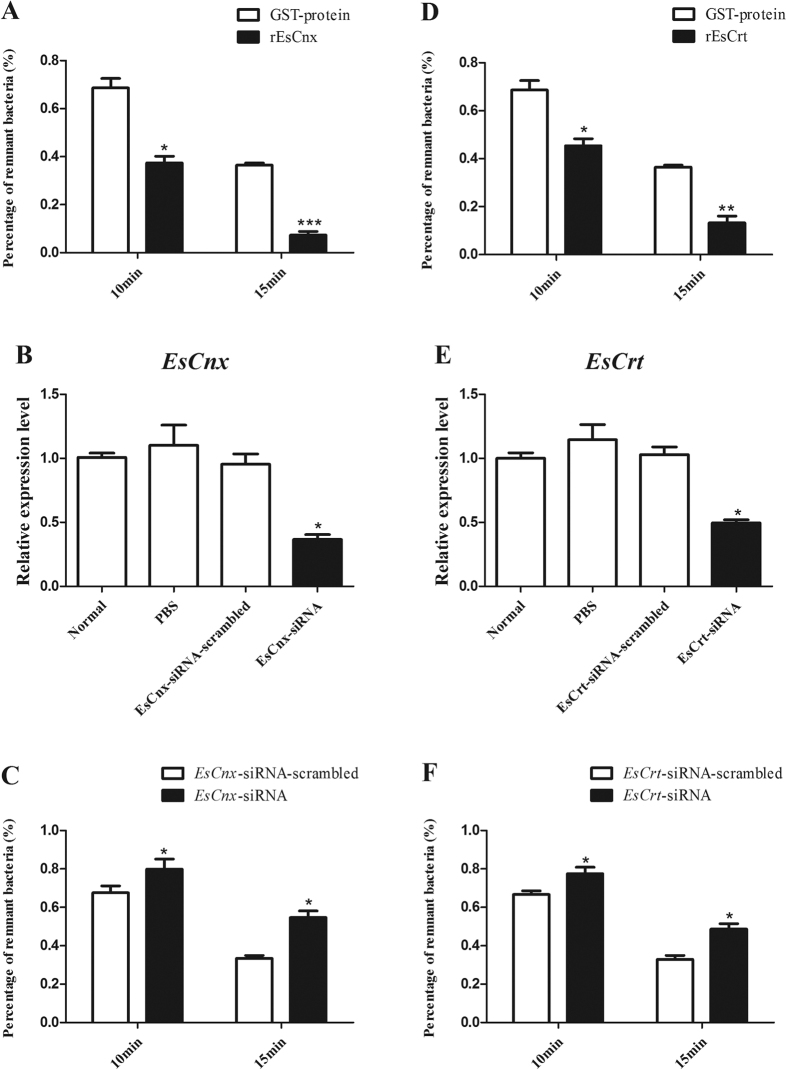
Effect of rEsCnx and rEsCrt in *V. parahaemolyticus* clearance activities in crab. rEsCnx (**A**) or rEsCrt (**D**) enhanced the clearance of *V. parahaemolyticus in vivo*. *V. parahaemolyticus* clearance assay was conducted by pre-coating the injected bacteria with rEsCnx, rEsCrt, or GST-tag protein. The number of remnant bacteria was counted at 2, 10, or 15 min post-injection. The percentage of remnant bacteria was calculated as follows: percentage of remnant bacteria = (number of bacteria at 10 min or 15 min/number of bacteria at 2 min) × 100. RNAi of *EsCnx* (**B**) or *EsCrt* (**E**) suppressed bacterial clearance in hemocytes. The mRNA transcription level of *EsCnx* 24 h after injection of *EsCnx*-siRNA or *EsCnx*-siRNA-scrambled, as revealed by qRT-PCR analysis. The mRNA transcription level of *EsCrt* 24 h after injection of *EsCrt*-siRNA or *EsCrt*-siRNA-scrambled was revealed by qRT-PCR. The clearance rate of *V. parahaemolyticus* in crabs was impaired by silencing *EsCnx* (**C**) or *EsCrt* (**F**) through RNAi. Two groups of crabs were injected with *EsCnx*-siRNA or *EsCrt*-siRNA-scrambled (control), and clearance assay was conducted 24 h post-injection.

Given that rEsCnx and rEsCrt can bind to the crab pathogen *V. parahaemolyticus*, we determined whether or not rEsCnx and rEsCrt affect the bacterial clearance rate in crabs. The bacterial clearance experiment showed that *V. parahaemolyticus* was cleared more rapidly in the hemolymphs of rEsCnx and rEsCrt injection groups compared with rArf and GST control groups (10 and 15 min, respectively) (Fig. 6A,D).

Clearance assays were also conducted after knockdown of *EsCnx* or *EsCrt*. *EsCnx* or *EsCrt* expression significantly decreased 24 h after injection of *EsCnx*-siRNA or *EsCrt*-siRNA (Fig. 6B,E); moreover, the clearance rate of *V. parahaemolyticus* in the crab significantly decreased after knockdown of *EsCnx* or *EsCrt* (Fig. 6C[Fig f6]F). These findings indicate that *EsCnx* and *EsCrt* play important functions in clearing *V. parahaemolyticus*.

## Discussion

Cnxs and Crts, which are important chaperones in the ER, are involved in translocation, protein folding, and quality control of newly synthesized polypeptides^4^. The roles of Cnxs and Crts in the innate immune system have been investigated in crustaceans. For instance, *Crt* was detected in the ovary and in various molting stages of Chinese white shrimp (*Fenneropenaeus chinensis*). When a host is exposed to heat shock or challenged by white spot syndrome virus (WSSV), Crt is rapidly cleaved and the shrimp initiates a defense reaction against the stimuli^32^. In crayfish, the C1q-binding proteins Crt and gC1qR, which are highly conserved ubiquitous proteins, can respond to viral infection by forming a complex in the cytoplasm, thereby preventing apoptosis^37^. Another Crt associated with immune defenses against *V. anguillarum* and WSSV was purified from the hemocytes of ridge tail white prawn (*Exopalaemon carinicauda*)^38^. A novel MjCnx protein was also detected in the cytoplasm and on the membrane of hemocytes in kuruma shrimp (*M. japonicas*); MjCnx protein not only enhances the clearance of *V. anguillarum in vivo* but also promotes phagocytic efficiency of hemocytes^39^. However, information on the function of Cnx and Crt in crab immunity remains unavailable^40^. Detecting Cnxs and Crts from crabs and investigating their immune mechanism can elucidate the functional diversity of crustacean Cnxs and Crts.

In this study, *EsCnx* and *EsCrt* were identified from *Eriocheir sinensis*. *EsCnx* and *EsCrt* were primarily distributed in hemocytes, hepatopancreas, and intestine; the expression levels of these proteins were altered by challenge with polysaccharides and microorganisms. Moreover, the mRNA transcripts of *MjCnx* from *M. japonicas*^39^ and *EcCrt* from *E. carinicauda*^38^ were upregulated in several tissues challenged with *V. anguillarum*. Hemocytes are important tissues involved in crustacean immunity and comprise many immune-related genes^41^. As the counterpart of the insect fat body and mammalian liver, the hepatopancreas is important for the humoral immune responses of crustaceans^42^. The arthropod alimentary tract is the first defense barrier against pathogen invasion^43^,^44^; furthermore, the intestine, as a crucial part of the alimentary canal, plays an essential role in crab immunity. These results suggest that *EsCnx* and *EsCrt* are involved in crab antibacterial immunity.

Since the discovery of Cnx and Crt in mammals^12^,^30^, the functions of these two proteins have been the subject of most studies. Cnx is a lectin chaperone, whereas Crt is a lectin-like chaperone^12^,^23^. In addition to the chaperone activity, Cnx and Crt also have nonchaperone fuctions such as phagocytosis and lectin activity^21^,^39^. In this study, crab rEsCnx and rEsCrt showed the lectin activities of binding diverse bacteria and several polysaccharides. The lectin activities of shrimp rMjCnx were also reported^39^. Lectins, which are pattern-recognition proteins, play crucial functions in recognizing and eliminating pathogens in innate immunity^45^. So, crab rEsCnx and rEsCrt may play an important role in recognizing and binding bacteria; this phenomenon is the first line of innate immune defense^46^,^47^.

Furthermore, we assumed that rEsCnx and rEsCrt can accelerate clearance of invading *V. parahaemolyticus*, which is a Gram-negative bacterium and an extremely virulent and prevalent pathogen that affects crab aquaculture^48^. Overexpression of rEsCnx or rEsCrt can enhance clearance of *V. parahaemolyticus in vivo* in crabs and the clearance of bacteria was impaired after knockdown of *EsCnx* or *EsEsCrt*. The rMjCnx also accelerate the removal of bacteria in shrimp^39^. It was also reported that rMjCnx can promote phagocytosis of bacteria^39^. Invertebrates exhibit effective innate immune responses, including humoral and cellular responses^49^. Cellular immune responses involve different types of hemocytes, which participate in pathogen clearance through phagocytosis of microorganisms^50^. Recognition of pathogen is the first step of phagocytosis and it needs the participation of receptors. In human, three types of receptors including pattern-recognition receptors (PRRs), opsonic receptors, and apoptotic corpse receptors are involved in phagocytosis^51^. The broad binding spectrum of rEsCnx and rEsCrt toward diverse bacteria and pathogen-associated molecular patterns (PAMPs) suggest that EsCnx and EsCrt may serve as phagocytic receptors.

In conclusion, this work is the first to characterize *EsCnx* and *EsCrt* isolated from *E. sinensis*. *EsCnx* and *EsCrt* expression levels are regulated by polysaccharide or bacterial challenge. Moreover, rEsCnx and rEsCrt proteins not only can bind to various bacteria and different polysaccharides (LPS and PGN) but also facilitate clearance of *V. parahaemolyticus* in crabs. Our results suggest that *EsCnx* and *EsCrt* are involved in anti-bacterial immunity in *E. sinensis*.

## Materials and Methods

### Cloning of full-length cDNAs of *EsCnx* and *EsCrt*

Two expressed sequence tags were obtained from the hepatopancreas of *E. sinensis* by using Illumina’s Solexa Sequencing Technology in the Chinese National Human Genome Center in Shanghai. Two pairs of gene-specific primers (*EsCnx*-F and *EsCnx*-R, as well as *EsCrt*-F and *EsCrt*-R) were designed based on the obtained cDNA sequences of *EsCnx* and *EsCrt* to clone their full-length sequences (the primer sequences are shown in Table 1).

### Sequence analysis

The cDNA sequences and deduced protein sequences of EsCnx and EsCrt were analyzed using the BLAST algorithm of the National Center for Biotechnology Information (http://blast.ncbi.nlm.nih.gov/Blast.cgi) and the Expert Protein Analysis System (http://web.expasy.org/translate/). Signal peptide and putative domains were predicted using the Simple Modular Architecture Research Tool (http://smart.embl-heidelberg.de/). Molecular weights and isoelectric points of EsCnx and EsCrt were determined using ExPASy (http://web.expasy.org/compute_pi/). Moreover, MEGA 5.05 was used to produce an unrooted phylogenetic tree based on the deduced full amino acid sequences of EsCnx and EsCrt and other related proteins by using the neighbor-joining method^52^. One thousand bootstraps were selected for the neighbor-joining tree to assess reliability. MEGA 5.05 and GENDOC were also used to perform multiple sequence alignments.

### Animals, polysaccharides, and microbes

Adult Chinese mitten crabs were purchased from a wet market (Nanjing, Jiangsu Province, China) and housed in filtered, aerated freshwater at 20–25 °C for a week before processing. LPS (*E. coli* Serotype 055:B5) and PGN (*M. luteus*) were obtained from Sigma (St. Louis, MO, USA). *S. aureus*, *M. luteus*, *B. subtilis*, *B. megaterium*, *A. hydrophila*, *V. parahaemolyticus*, *V. anguillarum*, and *E. coli* were purchased from the Microbial Culture Collection Center (Beijing, China) and grown in LB broth at 37 °C.

### Tissue collection and challenge with polysaccharides and microorganisms

Various tissues (hemocytes, heart, hepatopancreas, gills, muscle, intestine, nerve, and eyestalk from five adult crabs as parallel samples) were collected to determine the tissue distribution of *EsCnx* and *EsCrt* transcripts. The hemolymph was withdrawn using a 1 mL sterile syringe containing an equal volume of anticoagulant solution (ACD-B)^53^. The samples were immediately centrifuged at 800 × *g* at 4 °C for 15 min to collect hemocytes. A total of 150 crabs in the bacteria-challenged groups were used in the experiment. The crabs were injected with 50 μL of LPS (0.5 μg/μL), PGN (0.5 μg/μL), live *S. aureus* (3 × 10^7^ cells), or *V. parahaemolyticus* (3 × 10^7^ cells) in PBS. Five individual crabs were randomly sampled at 2, 6, 12, and 24 h in the challenge groups during the experiment. In addition, five untreated crabs were used as the untreated control group in the microorganism challenge. Hepatopancreas organs were collected from untreated control and microorganism-challenged treatment groups (LPS, PGN, *S. aureus*, and *V. parahaemolyticus*). All these tissue samples were stored at −80 °C for subsequent RNA extraction.

### Total RNA isolation and cDNA synthesis

Total RNA was extracted from the tissues according to the manufacturer’s instructions (Spin-column, BioTeke, Beijing, China). First-strand cDNA was obtained using PrimeScript First-strand cDNA synthesis kit with Oligo dT Primer from Takara (Dalian, China). The cDNA mixture was diluted 10 times with PCR-grade water and then stored at −80 °C for qRT-PCR analysis.

### Real-time PCR analysis of mRNA expression

The mRNA expression levels of *EsCnx* and *EsCrt* in different tissues and their temporal expression in the hepatopancreas of crabs after the treatments were measured by SYBR Green fluorescent RT-PCR. Two pairs of specific primers (*EsCnx*-RT-F and *EsCnx*-RT-R, as well as *EsCrt*-RT-F and *EsCrt*-RT-R) were used to amplify the corresponding products of *EsCnx* and *EsCrt*. The PCR products were sequenced to verify the specificity of qRT-PCR. Two *glyceraldehyde-3-phosphate dehydrogenase* (*GAPDH*) primers, namely, *EsGAPDH*-RT-F and *EsGAPDH*-RT-R (Table 1), were used for amplifying the 268-bp fragment as an internal control to verify successful transcription and to calibrate the cDNA template for corresponding samples. All samples were analyzed in triplicate in the qRT-PCR analysis. The expression levels of *EsCnx* and *EsCrt* were analyzed using the comparative CT method^54^; the discrepancy between the CT for *EsCnx* or *EsCrt*, and *GAPDH* (ΔCT) was calculated to normalize the variation in the amount of cDNA in each reaction. Moreover, *EsCnx* and *EsCrt* expression levels were calculated by 2^−^ΔΔ^CT^. The obtained data were statistically analyzed using an unpaired sample t-test. Significant difference was accepted as P < 0.05.

### Recombinant expression and purification of EsCnx and EsCrt in *E. coli*

The cDNA fragments encoding the Crt domains of EsCnx and EsCrt were amplified using the following primers: *EsCnx*-ex-F and *EsCnx*-ex-R, as well as *EsCrt*-ex-F and *EsCrt*-ex-R (Table 1), with *EcoR* I and *Not* I sites. The amplified products were cloned into pGEX-4T-1 vector (Novagen) and then transformed into competent *E. coli* Rosetta (DE3) cells (TransGen Biotech). The positive clones were screened through PCR reaction using specific primers and then confirmed by nucleotide sequencing. The cells were grown at 37 °C in LB medium under agitation until an OD600 of 0.6–0.8. Recombinant expression was induced by addition of isopropyl-b-D-1-thiogalactopyranoside (IPTG, 0.5 mmol L^−1^). The samples were then incubated for another 4 h under the same conditions. rEsCnx and rEsCrt were examined by SDS polyacrylamide gel electrophoresis (SDS-PAGE). In addition, rEsCnx and rEsCrt with a glutathione S-transferase (GST) tag and GST protein was purified using glutathione Sepharose 4B chromatography (Gen-Script, USA) according to the manufacturer’s instructions.

### Western blot analysis of localization and expression profiles of EsCnx and EsCrt proteins

Antisera for EsCnx and EsCrt were provided by Dr. Xian-Wei Wang (Shandong University)^39^,^55^. The heart, hepatopancreas, gills, muscle, intestine, nerve, and eyestalk of healthy crabs were homogenized in a buffer solution (50 mM Tris–HCl at pH 7.5, 150 mM NaCl, 3 mM EDTA, and 1 mM PMSF). The solution was then centrifuged at 10,000 × *g* for 10 min at 4 °C to collect the supernatant. The hemolymph of normal or challenged crabs was prepared for analysis. The protein concentration was determined using the method described by Bradford. For Western blot analysis, each sample (100 μg protein) separated by 12.5% SDS-PAGE gel was transferred onto a pre-wetted nitrocellulose membrane in electro blotting buffer under a constant current of 80 V for 100 min. The membrane was blocked in 5% non-fat dried milk in TBS (10 mM Tris–HCl, pH 7.5, 150 mM NaCl) at room temperature for 2 h. Subsequently, the membrane was incubated with 1/1000 diluted antiserum to EsCnx or EsCrt in TBS at 4 °C overnight and washed three times with TBST (10 mM Tris–HCl, pH 7.5, 150 mM NaCl, and 0.05% Tween-20) for 10 min. The membranes were then incubated with peroxidase-conjugated goat anti-rabbit IgG (1/5000 diluted in TBS) at room temperature for 2 h and then washed three times with TBST for 10 min. Protein bands were stained with 3,3-diaminobenzidine for 10 min, and the reaction was terminated by washing with distilled water.

### Immunofluorescence

Healthy crab was swabbed with 75% ethanol, and hemolymph was collected with an equal volume of ACD-B^53^. The diluted hemolymph was centrifuged at 300 × *g* at 4 °C for 10 min to collect the cells, which were washed two times in culture medium. The cells were resuspended gently in 2 mL culture medium, and 0.2 mL aliquots were seeded into 2 mL of culture medium on 12-well dishes with glass coverslips and then incubated at 28 °C. After attachment to the coverslips, the cells were washed three times with PBS. Paraformaldehyde solution (4%) in PBS was used to fix the cells for 30 min at room temperature. The cells were then washed in PBS three times and treated with 0.5% Triton X-100 solution for 10 min. After washing three times with PBS, the cells were treated with 5% BSA, blocked for 2 h, and then incubated with 1/1000 diluted rabbit antiserum to EsCnx or EsCrt in PBS at 4 °C overnight. The cells were washed four times with PBST and then incubated with Andy Fluor^TM^ 594 Goat Anti-Rabbit IgG (H+L) Antibodies (Beyotime, China) for 1 h. After washing four times with PBST, the cells were incubated with 500 μL of 25 μM DiOC6(3) (Beyotime, China) diluted in PBS at 37 °C for 10 min. The plates were washed as described above, and the cells were mounted on glass slides containing ProLong gold antifade reagent with DAPI (Invitrogen). After final washing, the cells were incubated in PBS and observed under a confocal laser scanning microscope.

### Microbial binding assay

The binding assay was performed as described earlier^56^. Briefly, microorganisms were collected and resuspended in TBS to OD600 of 1.0 and then incubated with recombinant EsCnx and EsCrt (600 μg/mL, 50 μL) for 30 min with gentle rotation at room temperature. The microorganisms were pelleted, washed four times with TBS, and then loaded onto 12.5% SDS-PAGE gel. The bound protein was detected by Western blot analysis using anti-GST antiserum. For the control, bacterial cells were incubated with rArf (600 μg/mL) and GST-tagged proteins and then subjected to the same treatments.

### Direct binding to PAMPs

ELISA assay was performed to detect the direct binding of EsCnx and EsCrt to LPS and PGN, as described previously^56^. LPS (50 μL) or PGN (80 μg/mL) was used to coat a 96-well microtiter plate. The plate was then incubated at 37 °C overnight, heated at 60 °C for 30 min, and then blocked with 200 μL/well BSA (600 μg/mL) in TBS for 2 h at 37 °C. After washing four times with TBS (200 μL/well), the purified EsCnx, EsCrt, or GST (control) was diluted in TBS containing 0.1 mg/mL BSA to achieve different concentrations (0–50 μg/mL) and added to each well (50 μL/well). Subsequently, the wells were incubated for 3 h at room temperature. The plates were then washed as described above, and anti-GST antiserum (1/2000 dilution) was added to the wells (100 μL/well). After 1 h of incubation at 37 °C, the wells were washed as described above. Approximately 100 μL of peroxidase-conjugated goat anti-rabbit IgG (1/5000 dilution) was added and incubated at 37 °C for 1 h. After final washing, color was enhanced by adding 0.01% 3,3′,5,5′-tetramethylbenzidine (Sigma) liquid substrate in citric acid–Na_2_HPO_4_ buffer. The reaction was terminated by adding 2 M H_2_SO_4_, and the absorbance was read at 450 nm. The binding assay was performed three times.

### *V. parahaemolyticus* clearance assay

Overnight-cultured *V. parahaemolyticus* was washed and resuspended in PBS. *V. parahaemolyticus* (approximately 3 × 10^7^ cells) was incubated with rEsCnx or rEsCrt (600 μg/mL, 500 μL) at 28 °C for 30 min with rotation. In addition, *V. parahaemolyticus* was incubated with GST (600 μg/mL, 500 μL) as a control. Two groups of mixtures (50 μL each) were injected into the crabs. The hemolymph (500 μL) of three crabs was collected at different post injection times (2, 10, and 15 min). After serial dilution with PBS, the samples (30 μL) were plated onto LB broth plates. The plates were incubated overnight at 37 °C, and the number of bacterial clones in the plate was counted thereafter. Subsequently, the percentage of residual bacteria (i.e., (number of bacteria in plasma at 10 or 15 min/number of bacteria in plasma 2 min after injection) × 100) was determined.

### *V. parahaemolyticus* clearance assay after siRNA interference

Small interfering RNAs (siRNAs), specifically those targeting *EsCnx* or *EsCrt*, were synthesized using an *In vitro* Transcription T7 Kit (Takara, Japan). *EsCnx*-siRNA and *EsCrt*-siRNA were used, and the siRNA sequence was scrambled to generate the control *EsCnx*-siRNA-scrambled or *EsCrt*-siRNA-scrambled (Table 1). Approximately 20 μg of siRNA (or siRNA-scrambled) was injected into the foot of the third appendage at a volume of 50 μL per crab. At 12 h after injection, the siRNA or siRNA-scrambled (20 μg) was injected into the same crab. At 24 h after the last injection, the total RNA of hemocytes from the two groups of crabs was isolated and used to detect the efficiency of RNAi through qRT-PCR. The remaining crabs were used for the clearance assay using the method described above with some modifications. In the modified clearance assay, 25 μL of *V. parahaemolyticus* (approximately 3 × 10^7^ cells), which was not incubated with the recombinant protein, was injected into the crab. The assays described earlier were biologically repeated three times.

## Additional Information

**How to cite this article**: Huang, Y. *et al*. Two endoplasmic reticulum proteins (calnexin and calreticulin) are involved in innate immunity in Chinese mitten crab (*Eriocheir sinensis*). *Sci. Rep*. **6**, 27578; doi: 10.1038/srep27578 (2016).

## Supplementary Material

Supplementary Information

## Figures and Tables

**Table 1:** Primer sequences used in this study.

Primers name	Sequences (5′–3′)
EsCnx-F	GTGACTGACAATACGGCTGAGGCTGACC
EsCnx-R	CCACTCCTTCCTTCTTTGCCTGTGACTTG
EsCrt-F	CCCCGAGTACACCCCTGACACAGAAATCT
EsCrt-R	GCTCTTCACCTGCCACAAGTCCAAACCGA
EsCnx-RT-F	AAATGGATGGTGAATGGGAGGC
EsCnx-RT-R	TTGGGGATGGTGCGAGGTT
EsCrt-RT-F	GGAGAGCAAGGAGGATGAGGAG
EsCrt-RT-R	AAGGGGATTGACTGAGGAAAGATTA
EsGAPDH-RT-F	CTGCCCAAAACATCATCCCATC
EsGAPDH-RT-R	CTCTCATCCCCAGTGAAATCGC
EsCrt-ex-F	CCGGAATTCGCTTATGTCATGGAAACATTT
EsCrt-ex-R	TACTCAGCGGCCGCTCCACAATAAGGTTATCAAAAAGG
EsCrt-ex-F	CCGGAATTCGTGTTCTTCCAGGAGACCTTC
EsCrt-ex-R	TACTCAGCGGCCGCTCAATGAGGAAGTTGTCGAA
*EsCnx*-siRNA	GCTGGCTGCAGACACTTAT
*EsCnx*-siRNA-scrambled	ATTGCAGCTGGCAAGCCTT
*EsCrt*-siRNA	GGTGTGAAAGAACCACAAT
*EsCrt*-siRNA-scrambled	TGAGGACATGACATGAAAC

*EcoR* I and *Not* I sites are underlined.
