# Supplementary material for: Two endoplasmic reticulum proteins (calnexin and calreticulin) are involved in innate immunity in Chinese mitten crab (Eriocheir sinensis)
Source: Sci Rep. 2016 Jun 9;6:27578. doi: 10.1038/srep27578 (PMC4899775; doi:10.1038/srep27578)

**Title page**

**Two endoplasmic reticulum proteins (calnexin and calreticulin) are involved in innate immunity in Chinese mitten crab (*Eriocheir sinensis*)**

Ying Huang^1^, Kaimin Hui^1^, Min Jin^3^, Shaowu Yin^1,2^, Wen Wang^1*^, Qian Ren^1*^

1, Jiangsu Key Laboratory for Biodiversity & Biotechnology and Jiangsu Key Laboratory for Aquatic Crustacean Diseases, College of Life Sciences, Nanjing Normal University, Nanjing 210046, China

2, Co-Innovation Center for Marine Bio-Industry Technology of Jiangsu Province, Lianyungang, Jiangsu 222005, PR China

3, MOE Key Laboratory of Aquatic Product Safety/State Key Laboratory of Biocontrol, School of Marine Sciences, Sun Yat-sen University, Guangzhou, China

* Corresponding author: Prof Qian Ren

Tel: 86-25-85891955

E-mail: [renqian0402@126.com](mailto:renqian0402@126.com)

Prof Wen Wang

Tel: 86-25-85891955

E-mail:njnuwang@263.net

Ying Huang and Kaimin Hui contributed equally to this paper.

**Fig 1S. Nucleotide and deduced amino acid sequences of EsCnx (A) and EsCrt (B) cDNAs from *Eriocheir sinensis*.** Start codon (ATG) and stop codon (TAG) are shown in red. The signal peptide of EsCnx and EsCrt are italicized. The shaded sequences denote the calreticulin domain. The transmembrane region is underlined, and the coiled region is double underlined.

**Fig 2S. Multiple alignments of EsCnx containing calnexin (Cnx) from other species (A) or EsCrt containing other calreticulin (Crt) (B).** Dashes (–) indicate gaps, black shadow indicates identical residues, and gray shadow indicates similar residues in the aligned amino acid sequences. MjCnx: Cnx from *Marsupenaeus japonicas* (AIF71174.1); PmCnx: Cnx from *Penaeus monodon* (ADO00931.1); SpCrt: Crt from *Scylla paramamosain* (AEN94572.1); CqCrt: Crt from *Cherax quadricarinatus* (AIW68605.1); CcCrt: Crt from *Cherax cainii* (AJO70188.1); CdCrt: Crt from *Cherax destructor* (AJO70005.1); PlCrt: Crt from *Pacifastacus leniusculus* (AEC50079.1); LvCrt: Crt from *Litopenaeus vannamei* (AFC34501.1); PcCrt: Crt from *Palaemon carinicauda* (AGJ03552.1); FcCrt: Crt precursor from *Fenneropenaeus chinensis* (ABC50166.1); PmCrt: Crt from *P. monodon* (ADO00927.1).

**Fig 3S. Phylogenetic tree analysis of EsCnx and other representative calnexin (Cnx) proteins.** *Marsupenaeus japonicas* Cnx: Accession No. AIF71174.1; *Penaeus monodon* Cnx: Accession No. ADO00931.1; *Tribolium castaneum* Cnx: Accession No. XP_975051.1; *Camponotus floridanus* Cnx: Accession No. EFN68342.1; *Camponutus floridanus* Cnx-like: Accession No. XP_011256487.1; *Aptenodytes forsteri* Cnx: Accession No. XP_009284137.1; *Bombyx mori* Cnx-like: Accession No. XP_004927743.1; *Pediculus humanus corporis* Cnx precursor: Accession No. XP_002431724.1; *Zootermopsis nevadensis* Cnx: Accession No. KDR07563.1; *Ixodes scapularis* Cnx: Accession No. XP_002401232.1; *Culex quinquefasciatus* Cnx: Accession No. XP_001850652.1; *Merops nubicus* Cnx isoform: Accession No. XP_008938002.1; *Tinamus guttatus* Cnx isoform: Accession No. XP_010216721.1; *Danio rerio* Cnx precursor: Accession No. NP_998613.1; *Acanthisitta chloris* Cnx: Accession No. KFP78131.1; *Gallus* Cnx precursor: Accession No. NP_001025791.2; and *Microplitis demolitor* Cnx-like: Accession No. XP_008548369.1.

**Fig 4S. Phylogenetic tree analysis of EsCrt and other representative calreticulin (Crt) proteins.** *Scylla paramamosain* Crt: Accession No. AEN94572.1; *Cherax quadricarinatus* Crt: Accession No. AIW68605.1; *Cherax cainii* Crt: Accession No. AJO70188.1; *Cherax destructor* Crt: Accession No. AJO70005.1; *Pacifastacus leniusculus* Crt: Accession No. AEC50079.1; *Litopenaeus vannamei* Crt: Accession No. AFC34501.1; *Palaemon carinicauda* Crt: Accession No. AGJ03552.1; *Fenneropenaeus chinensis* Crt precursor: Accession No. ABC50166.1; *Panaeus monodon* Crt: Accession No. ADO00927.1; *Bombus terrestris* Crt-like: Accession No. XP_003403200.1; *Plutella xylostella* Crt: Accession No. ADN06079.1; *Fopius arisanus* Crt: Accession No. XP_011311869.1; *Apis mellifera* Crt: Accession No. XP_006559569.1; *Bombus impatiens* Crt-like: Accession No. XP_003486363.1; *Apis florea* Crt-like: Accession No. XP_003692561.1; *Culex quinquefasciatus* Crt: Accession No. XP_001848824.1; *M. demolitor* Crt: Accession No. XP_008559929.1; *Polyrhachis vicina* Crt: Accession No. AFL48552.1; *Galleria mellonella* Crt: Accession No. BAB79277.1; *Papilio xuthus* Crt: Accession No. BAM17778.1; *Danaus plexippus* Crt: Accession No. EHJ72848.1; *Acromyrmex echinatior* Crt: Accession No. XP_011061049.1; and *Solenopsis invicta* Crt: Accession No. XP_011155626.

Fig S1


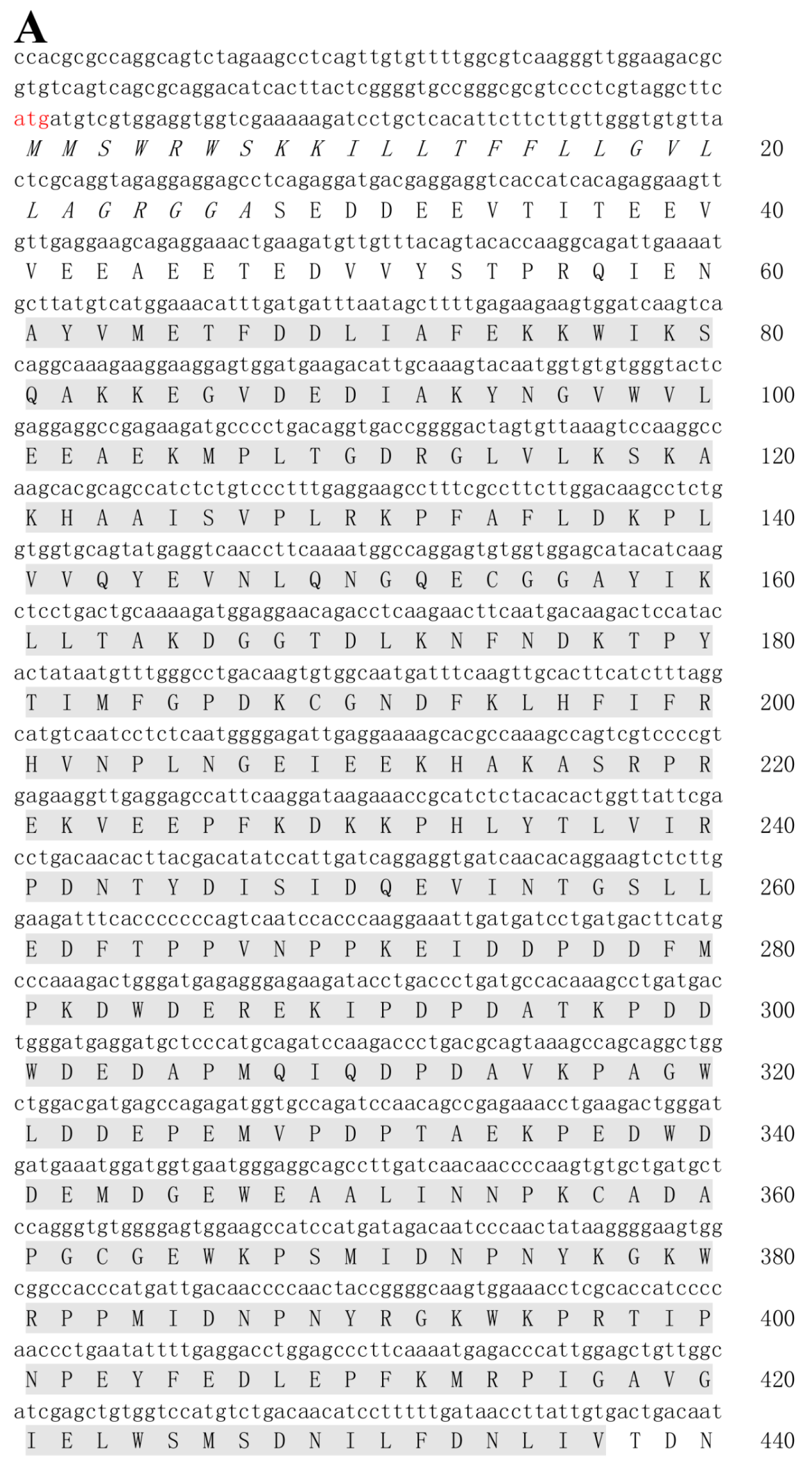


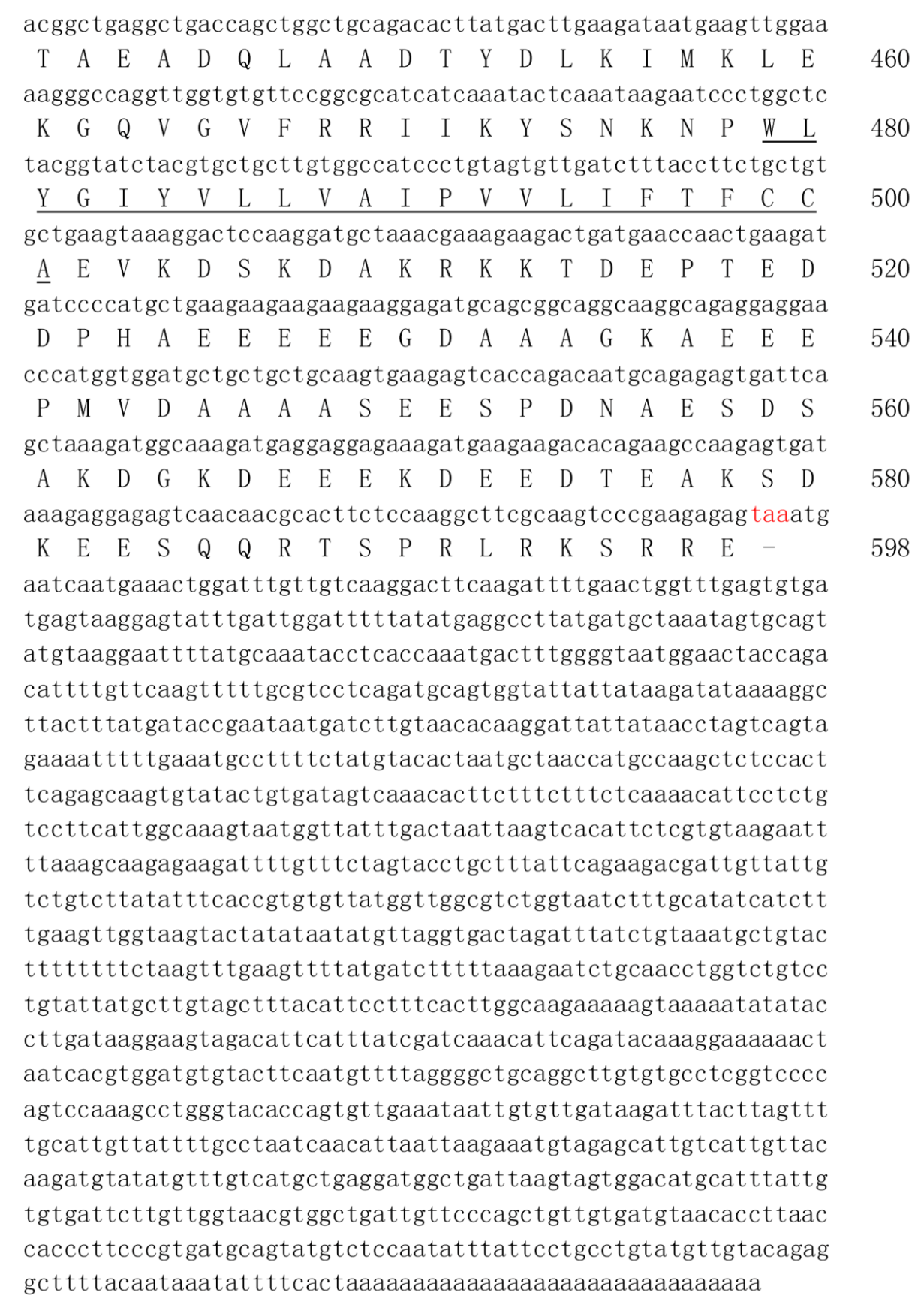


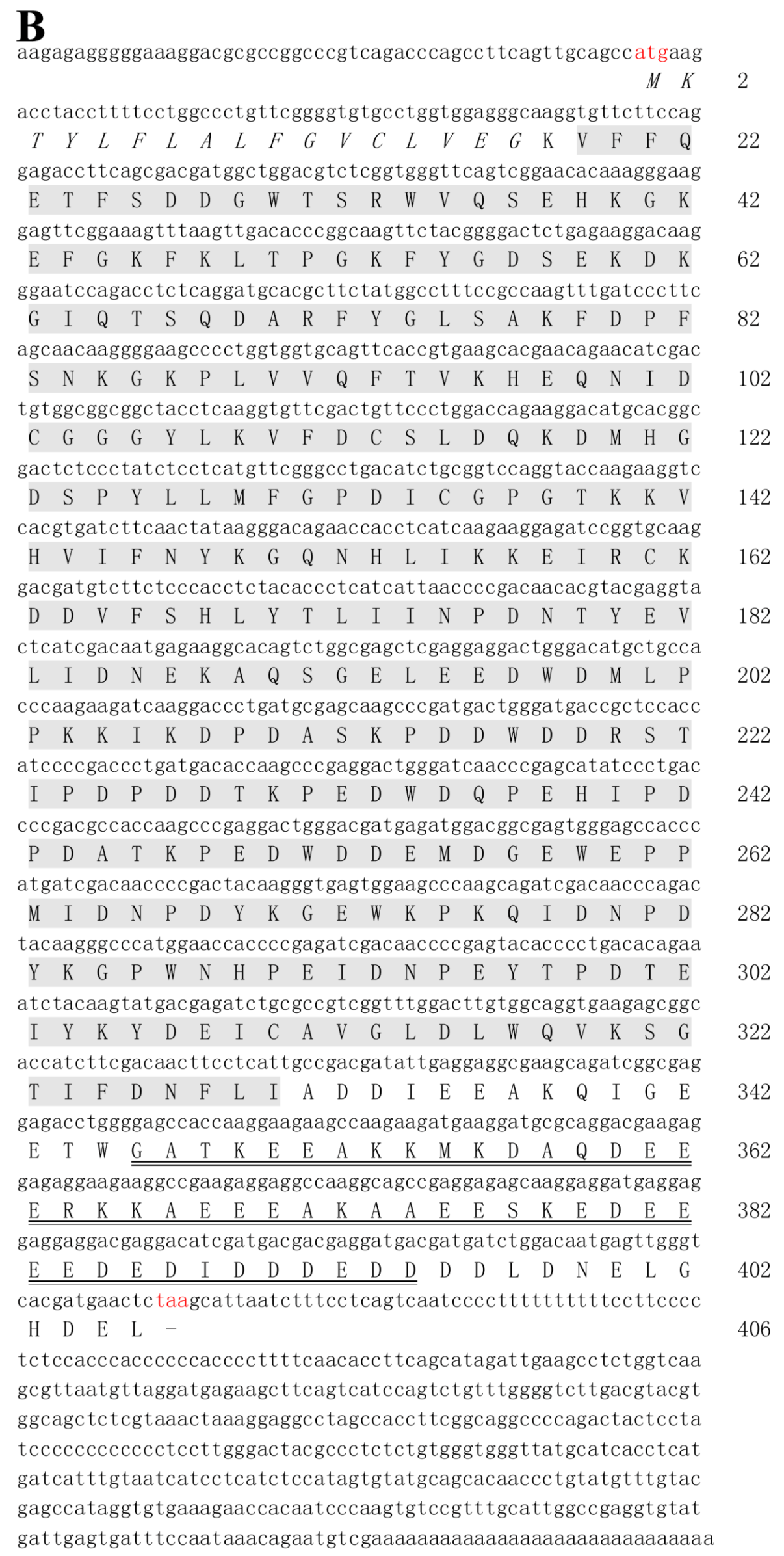


Fig S2


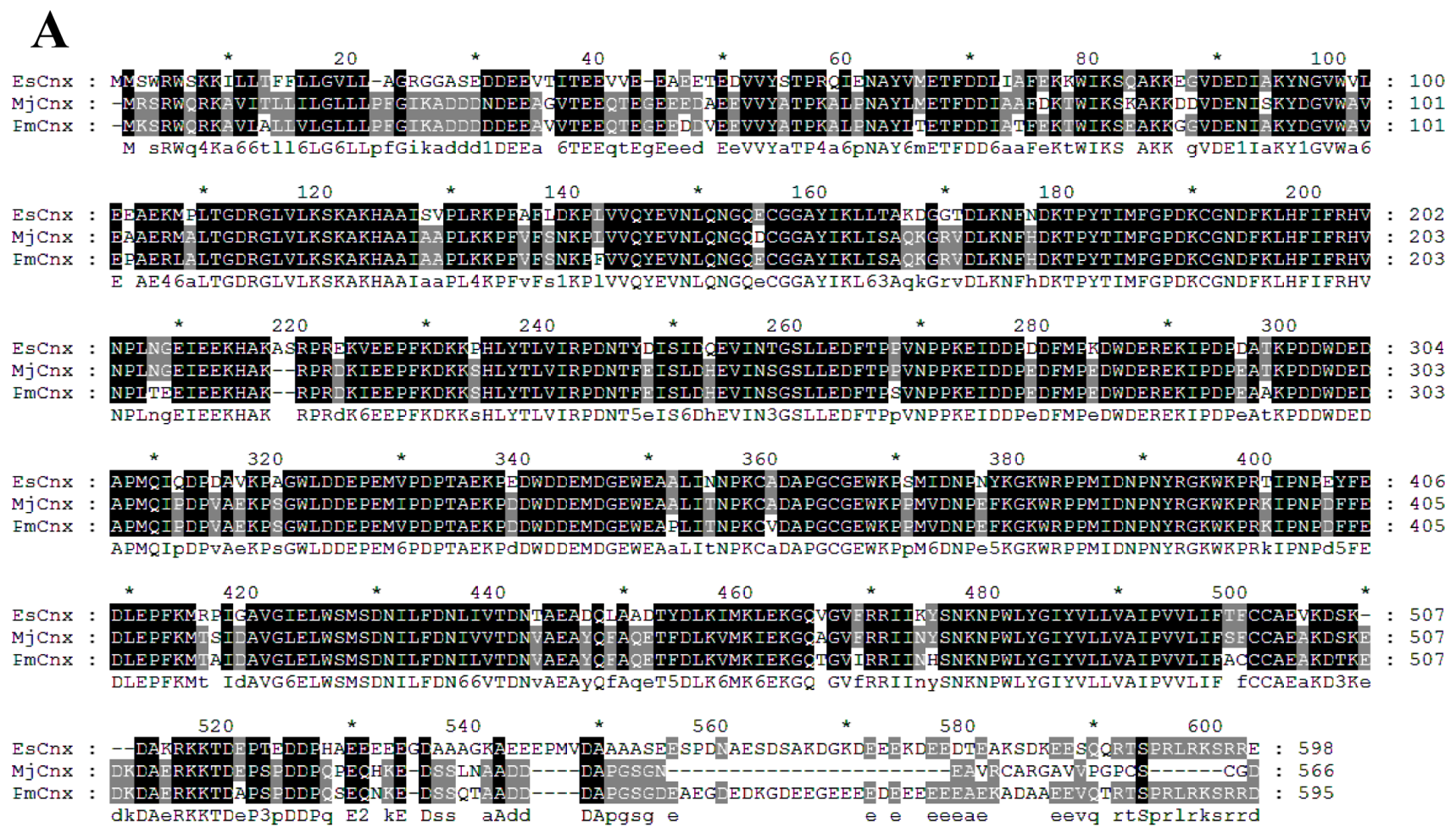


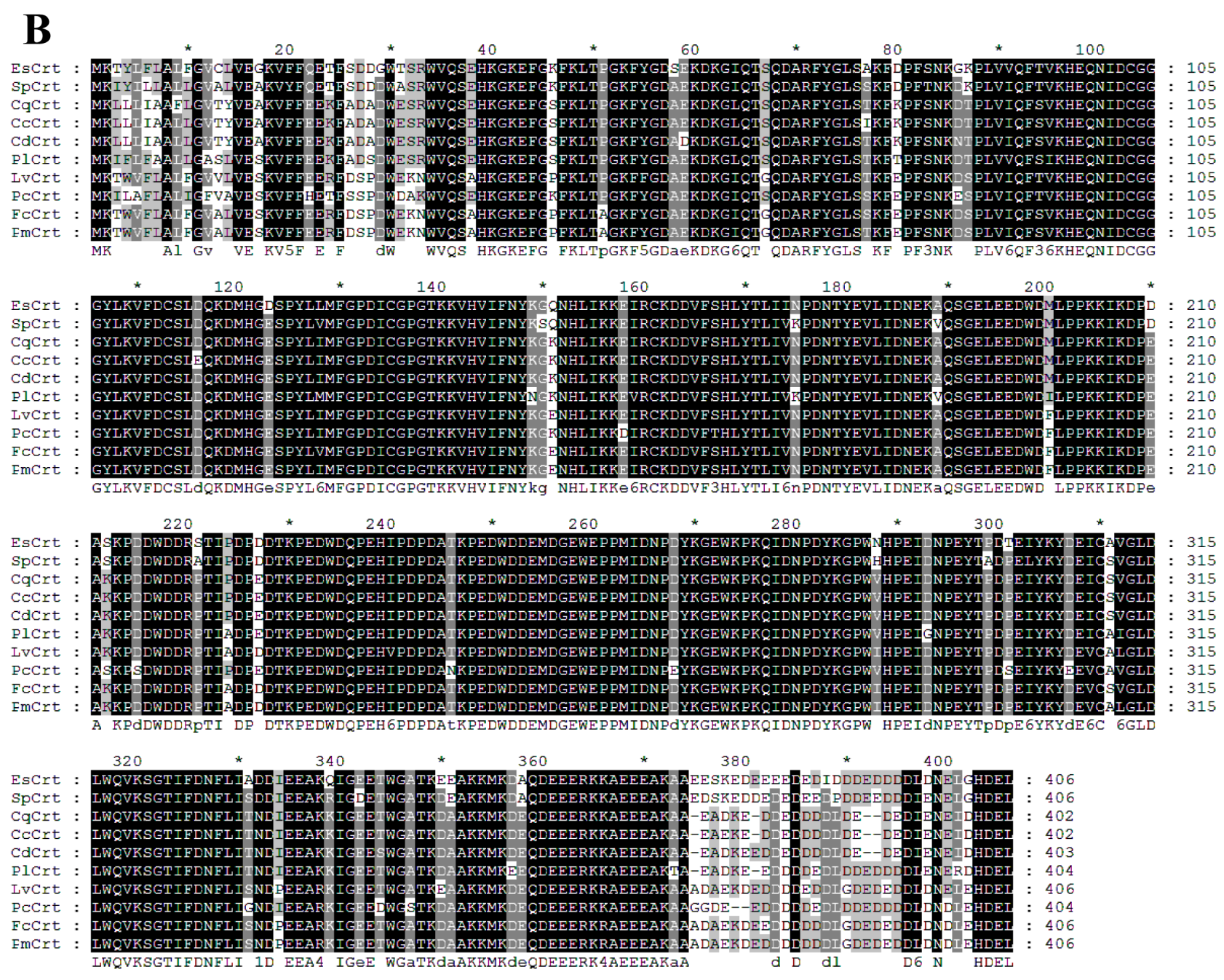


Fig 3S


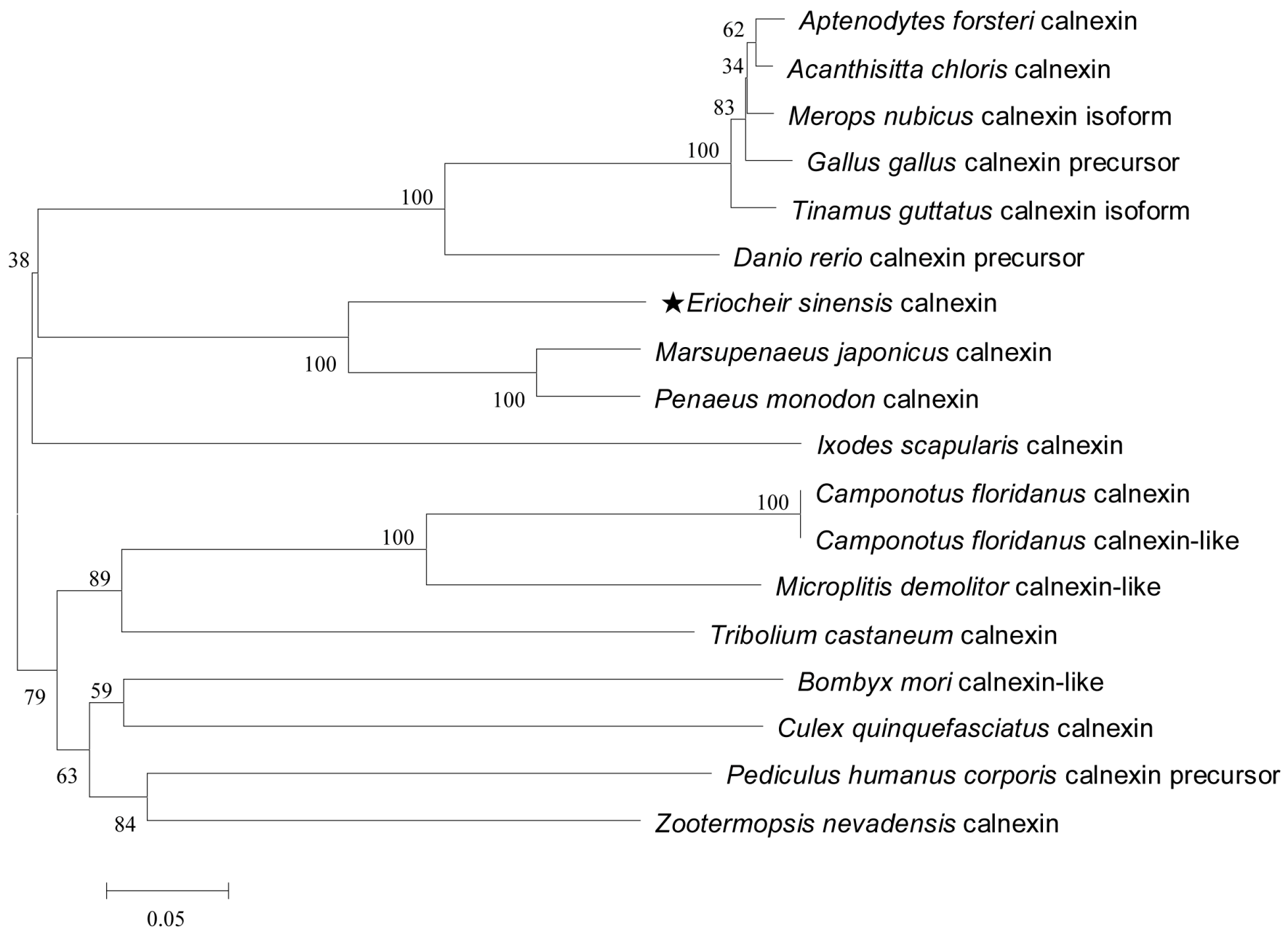


Fig 4S


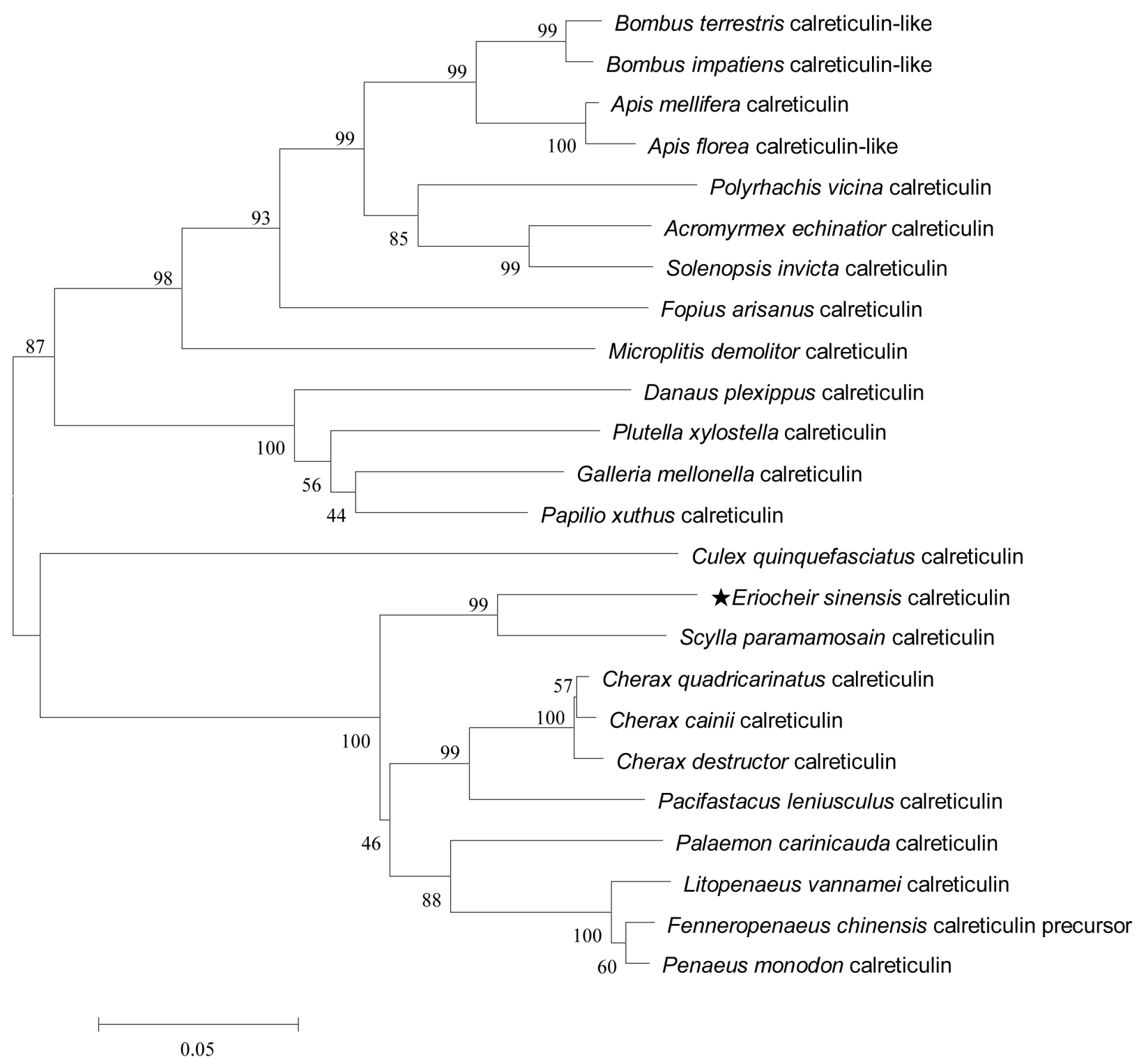

Supplement: Supplementary Information [file srep27578-s1.doc]
